# Intrinsic molecular subtypes of breast cancers categorized as HER2-positive using an alternative chromosome 17 probe assay

**DOI:** 10.1186/s13058-018-1005-z

**Published:** 2018-07-11

**Authors:** Neelam V. Desai, Vanda Torous, Joel Parker, James T. Auman, Gary B. Rosson, Cassandra Cruz, Charles M. Perou, Stuart J. Schnitt, Nadine Tung

**Affiliations:** 1 Department of Hematology-Oncology, Beth Israel Deaconess Medical Center and Harvard Medical School, 330 Brookline Ave, Shapiro 9, Boston, MA 02215 USA; 2 Department of Pathology, Beth Israel Deaconess Medical Center and Harvard Medical School, Boston, MA USA; 3 Department of Genetics and Lineberger Comprehensive Cancer Center, University of North Carolina at Chapel Hill, Chapel Hill, North Carolina USA; 4 Department of Pathology and Laboratory Medicine and Lineberger Comprehensive Cancer Center, University of North Carolina at Chapel Hill, Chapel Hill, North Carolina USA; 5 Department of Pathology, Dana-Farber/Brigham and Women’s Cancer Center and Harvard Medical School, Boston, MA USA

**Keywords:** HER2-positive breast cancer, PAM50 intrinsic subtype, HER2 alternative probe, ASCO-CAP guidelines

## Abstract

The 2013 update of the American Society of Clinical Oncology-College of American Pathologists (ASCO-CAP) human epidermal growth factor receptor 2 (HER2) testing guidelines recommend using an alternative chromosome 17 probe assay to resolve HER2 results determined to be equivocal by immunohistochemistry (IHC) or fluorescence in-situ hybridization (FISH). However, it is unclear if cases considered HER2-positive (HER2^+^) by the alternative probe method are similar to those classified as HER2^+^ by traditional IHC and FISH criteria and benefit the same from HER2-targeted therapies. We studied the clinical and pathologic features of all 31 breast cancers classified as HER2^+^ by the alternative probe method at our institution since 2013 and determined their PAM50 intrinsic molecular subtypes. For comparison, we analyzed 19 consecutive cases that were classified as HER2^+^ by traditional FISH criteria during the same time period. Thirty (97%) cancers in the alternative probe cohort were estrogen receptor (ER)-positive (ER^+^), while only 9/19 (47%) of traditional HER2 controls were ER^+^ (*p* = 0.0002). Sufficient tissue for intrinsic subtype analysis was available for 20/31 cancers in the alternative probe cohort and 9/19 in the traditional HER2^+^ group. None (0%) of the 20 alternative probe-positive cases were of the HER2-enriched intrinsic subtype, while 8/9 (89%) of those HER2^+^ by traditional FISH criteria were HER2-enriched (*p* = 0.0001). These findings suggest that breast cancers classified as HER2^+^ only by the alternative probe method are biologically distinct from those classified as HER2^+^ by traditional criteria, and raises questions as to whether or not they derive the same benefit from HER2-targeted therapies.

Approximately 15% of breast cancers are classified as human epidermal growth factor receptor 2 (HER2)-positive (HER2^+^) [1]. HER2 positivity confers an aggressive phenotype and was associated with poor long-term outcomes [2] until the incorporation of HER2-targeted agents into treatment which resulted in a 50% reduction in recurrence and a 30% improvement in survival [3, 4].

The two most common methods for HER2 testing are immunohistochemistry (IHC) to assess protein overexpression and fluorescence in-situ hybridization (FISH) to assess gene amplification. Single-probe FISH enumerates HER2 copies per nucleus. Dual-probe FISH includes both an HER2 probe and a chromosome 17 centromere probe, providing an HER2/CEP17 ratio in addition to absolute HER2 copy number [5]. Intrinsic molecular subtype analysis using gene expression profiling is not used clinically, but may better represent the inherent biologic heterogeneity of breast cancer than conventional biomarkers such as estrogen receptor (ER), progesterone receptor (PR), and HER2 [6, 7]. While all intrinsic molecular subtypes have been observed among clinically HER2^+^ breast cancers defined by traditional IHC/FISH criteria, the majority (67%) are classified as HER2-enriched, with ER-positive (ER^+^)/HER2^+^ cases being HER2-enriched 54% of the time [8]. In contrast, among clinically HER2-negative (HER2^–^) cases, only ~ 7% are of the HER2-enriched subtype [9]. Recent data suggest that the HER2-enriched molecular subtype may be a better predictor of response to HER2-targeted therapy than IHC or FISH assessments [10].

The American Society of Clinical Oncology (ASCO) and College of American Pathologists (CAP) updated their HER2 testing guidelines in 2013 [11]. It is now recommended that for HER2 equivocal cases (i.e., IHC 2+ or HER2 copies ≥ 4 and < 6 and HER2/CEP17 ratio < 2), FISH be performed using a probe for other genes on chromosome 17 to serve as the denominator to determine the HER2/chromosome 17 ratio. Genes such as retinoic acid receptor alpha (RARA), Smith-Magenis Syndrome (SMS), or tumor protein p53 (TP53) are located further away from HER2 than the centromere and are thus less likely to be co-amplified with HER2 than the centromere. Thus, an HER2/alternative chromosome 17 probe ratio of ≥ 2 may more accurately reflect HER2 gene amplification than the HER2/CEP17 ratio [12].

Using the alternative probe method has resulted in an increased number of breast cancers classified as HER2-positive [13]. However, whether or not these cancers have similar clinical and pathologic features or respond as well to HER2-targeted therapy as breast cancers defined as HER2-positive by traditional IHC/FISH criteria is unclear.

We identified all breast cancers at our institution since 2013 classified as HER2-positive only by the alternative probe method. We identified 31 such cases, and 20 of those had sufficient tissue for PAM50 intrinsic subtype analysis. As a comparison group, we selected 19 consecutive cases classified as HER2^+^ by traditional FISH criteria during the same period. We reviewed their clinical and pathologic features and determined intrinsic molecular subtype analysis using the PAM50 research-based assay as previously described [14] (Tables 1 and 2).

**Table 1:** Clinical and pathologic characteristics of breast cancer patients with positive HER2 alternative probe

Patient no.	Histology	Grade	ER/PR	HER2 IHC^a^	HER2 Copies	HER2/CEP17 ratio	HER2/P53 ratio	HER2 FISH repeated on a 2nd tumor specimen	Intrinsic subtype	TNM stage	Herceptin ± chemo given?	NAT, AT, MET	Herceptin ± chemo regimen^b^	If NAT, pathologic response?	Follow-up time^c^	Disease status
1	IDC	3	+/+	+ 2	4.15	1.1	2.2	Negative	Luminal A	pT1aN0	No	N/A	N/A	N/A	16 months	Remission
2	ILC	2	+/−	+ 2	4.47	1.5	2.9	Negative	Normal	cT2N1	No	NAT	ddAC-T	PR	42 months	Remission
3	IDC	3	+/+	+ 2	5.77	1.8	2.8	Not done	Luminal A	cT3N0	Yes	NAT	THP	CR	22 months	Remission
4^d^	IDC	3	+/+	+ 2	4.9	1.2	2.8	Not done	Luminal B	cT1cN1	Yes	NAT	ddAC-THP	PR	16 months	Remission
5	IDC	2	+/+	0–1+	4.67	1.5	2.1	Pos by alt probe	Luminal A	pT1aN0	Yes	AT	TH	N/A	24 months	Remission
6^e^	IDC	2	+/+	+ 3	4.51	1.3	2.6	Pos by alt probe	Luminal B	pT1bNx	Yes	AT	TCy-HP	N/A	15 months	Remission
7	IDLC	2	+/+	+ 2	4.88	1.5	3.0	Pos by conventional FISH^f^	Luminal A	pT2N2	Yes	AT	ddAC-THP	N/A	12 months	Remission
8	IDLC	3	+/+	+ 2	4.05	1.9	2.2	Not done	Luminal A	pT1cN1a	Yes	AT	TCy-H	N/A	35 months	Remission
9	IDC	3	+/−	+ 2	4.62	1.5	2.7	Negative	Luminal B	cT1cN1a	Yes	AT	TCH	N/A	33 months	Remission
10	IDC	2	+/+	+ 2	5.42	1.5	2.6	Not done	Luminal A	cT2N1a	Yes	AT	ddAC-THP	N/A	31 months	Remission
11	IDLC	3	+/+	+ 3	5.42	1.3	2.6	Not done	Luminal B	cT2N0	Yes	AT	ddAC-THP	N/A	18 months	Remission
12	IDLC	2	+/+	+ 2	4.45	1.5	3.3	Negative	Luminal A	pT1bN0	Yes	AT	TH	N/A	29 months	Remission
13	IDC	2	+/+	+ 2	4.12	1.2	2.1	Negative	Luminal A	cT2N1M1b	Yes	MET	capecitabine	N/A	19 months	Stable dz
14	IDC	1	+/−	+ 3	5.93	1.7	2.8	na	Luminal A	na	na	na	na	na	na	na
15	IDC	3	−/−	0–1+	5.56	1.2	2.3	Negative	NS	cT2N0	Yes	NAT	ddAC-THP as NAT, capecitabine as AT	PR	27 months	Remission
16	IDC	2	+/+	+ 3	5.83	1.6	3.2	Not done	NS	pT1bN0	Yes	AT	TH	N/A	40 months	Remission
17	IDC	2	+/+	0–1+	4.17	1.3	2.3	Not done	NS	pT1cN1mi	No	AT	TCy	N/A	26 months	Remission
18	IDLC	2	+/+	+ 2	4.72	1.2	3.3	Not done	NS	pT1bN0	Yes	AT	trastuzumab and endocrine tx	N/A	2 months	Remission
19	ILC	2	+/+	+ 2	4.37	1.6	2.7	Not done	NS	cTxNxM1b	Yes	MET	Various chemo + anti-HER2 tx	N/A	36 months	Died of disease
20	ILC	2	+/+	+ 2	4.62	1.5	2.3	na	NS	na	na	na	na	na	na	na
21	IDLC	1	+/+	+ 2	4.42	1.4	2.6	na	NS	na	na	na	na	na	na	na
22	IDC	3	+/+	+ 2	4.42	1.6	2.2	na	NS	pT2N2Mx	na	na	na	na		na
23	IDC	1	+/+	+ 2	4.45	1.6	2.1	na	NS	na	na	na	na	na	na	na
24	IDLC	3	+/+	0–1+	4.13	1.5	2.9	Not done	NS	pT1cN0	na	na	na	na	na	na
25	IDLC	3	+/+	+ 2	4.68	1.2	2.5	na	NS	na	na	na	na	na	na	na
26^d^	IDLC	2	+/+	0–1+	5.13	1.4	2.1	Not done	Luminal A	pT1cN0	No	N/A	N/A	N/A	9 months	Remission
27^d^	IDLC	2	+/+	+ 2	4.22	1.7	3.2	Positive by conventional FISH^f^	Luminal A	pT1bN0	Yes	AT	TH ×2 weeks, then T-DM1 ×3 doses	N/A	13 months	Remission
28	IDC	3	+/+	+ 2	5.17	1.9	2.2	Not done	Luminal A	pT1bN0	Yes	AT	TH	N/A	7 months	Remission
29	IDC	2	+/+	+ 2	4.79	1.4	2.5	Negative	Luminal A	pT2N1a	Yes	AT	ddAC-THP	N/A	6 months	Remission
30	IDC	2	+/+	+ 2	4.45	1.6	3.5	na	Luminal B	na	na	na	na	na	na	na
31	IDC	2	+/+	+ 3	5.07	1.3	2.6	na	Luminal A	na	na	na	na	na	na	na

^a^HER2 IHC: 0–1+ is negative, 2+ is equivocal, 3+ is positive

^b^All patients who received trastuzumab-based treatment received trastuzumab (H) for 1 year unless otherwise specified

^c^Follow up time defined as time in months from date of initial breast biopsy to date of last clinical follow-up

^d^Patients with oncotype Dx score: patient 4 score 29, patient 26 score 12, patient 27 score 21

^e^Patient had post-mastectomy recurrence and had history of prior ALND; so current nodal status could not be assessed

^f^Repeat HER2 testing for patient 7 showed HER2 copies of 4.53 and HER2/CEP17 ratio of 2.0 and for patient 27 showed HER2 copies of 6.5 and HER2/CEP17 ratio of 2.3

*AT* adjuvant therapy, *CR* complete response, *ddAC* dose-dense adriamycin + cyclophosphamide, *ER* estrogen receptor, *FISH* fluorescence in-situ hybridization, *H* herceptin (trastuzumab), *HER2* human epidermal growth factor receptor 2, *IDC* invasive ductal cancer, *IDLC* invasive cancer with ductal and lobular features, *IHC* immunohistochemistry, *ILC* invasive lobular cancer, *MET* therapy for metastatic disease, *N/A* not applicable, *na* not available, *NAT* neoadjuvant therapy, *NS* not sufficient for testing, *P* pertuzumab, *PR* partial response, *PR* progesterone receptor, *T* weekly paclitaxel ×12, *TC* taxotere (docetaxel) + carboplatin, *TCy* taxotere (docetaxel) + cyclophosphamide, *TNM* tumor node metastasis, *Tx* therapy

**Table 2:** Randomly selected consecutive HER2^+^ cases by traditional FISH criteria

Patient no.	Histology	Grade	ER/PR	HER2 IHC^a^	HER2 copies	HER2/CEP17 ratio	Intrinsic subtype	TNM stage	Herceptin ± chemo given?	NAT, AT, MET	Herceptin ± chemo regimen^b^	If NAT, pathologic response?	Follow-up time^c^	Disease status
1	IDC	3	+/+	+ 3	19.1	5.8	NS	pT1cN0	No; patient declined	N/A	N/A	N/A	9 months	Remission
2	IMPC	2	−/−	+ 3	17.6	4.8	NS	pT1bNx	No; not offered 2/2 age/comorbidities	N/A	N/A	N/A	4 months	Died of AAA rupture
3	IDC	3	+/+	+ 3	20.6	6.6	Luminal A	cT2N1M0	Yes	NAT & AT	NAT: T-DM1 + P on a trial, AT: THP	PR	25 months	Remission
4	IDC	3	+/+	+ 3	> 10	5.2	HER2-E	cT1cN0	Yes	NAT & AT	NAT: T-DM1 + P on a trial, then AT: docetaxel ×4 cycles + H ×1 year	CR	12 months	Remission
5	IDC	3	−/−	+ 3	> 20	1.0^d^	HER2-E	cT1cN0	Yes	NAT	THP	CR	18 months	Remission
6	IDC	2	−/−	+ 3	12.8	5.4	NS	cT2-3 N1	Yes	NAT	THP-ddAC	PR	5 months	Remission
7	IDC	3	−/−	+ 3	23.3	5.3	HER2-E	cT2N0	na	na	Na	na	na	na
8	IDC	3	−/−	+ 3	20.7	6.4	NS	pT1cNx	Yes	AT	TH	N/A	27 months	Remission
9	ILC	2	−/−	+ 3	> 6	5.6	NS	pT1aN0	Yes	AT	TH	N/A	25 months	Remission
10	IDC	3	−/−	+ 3	26.3	7.6	HER2-E	pT1bN0	Yes	AT	TH	N/A	30 months	Remission
11	IDC	3	−/−	+ 3	> 10	> 3.8	HER2-E	pT1bN0	Yes	AT	TH	N/A	21 months	Remission
12	IMPC	3	−/−	+ 3	19.85	7.4	HER2-E	pT1cN1a	Yes	AT	ddAC-TH	N/A	28 months	Remission
13	IDC	2	+/−	+ 3	37.2	6.8	NS	pT1bN0	Yes	AT	ddAC-TH	N/A	26 months	Remission
14	IDC	3	+/−	+ 3	6.88	3.2	NS	pT2N1a	Yes	AT	THP ×12 weeks, ddACX2 (stopped 2/2 SAE, 1 year H)	N/A	22 months	Remission
15	IDC	3	+/−	+ 3	> 20	9.5	NS	pT1aN0	Yes	AT	T-DM1 on a trial	N/A	20 months	Remission
16	IDC	3	+/−	+ 3	23	8.1	HER2-E	pT1bN1a	Yes	AT	ddAC-THP	N/A	17 months	Remission
17	IDC	3	+/+	+ 3	> 10	3	NS	T2N1M1b	Yes	MET	THP, AC, various	N/A	35 months	Alive, on tx
18	IDC	3	−/−	+ 3	26.7	9.4	HER2-E	na	na	na	na	na	na	na
19	IDC	2	+/−	+ 3	20.3	3.4	NS	cT3N1	na	na	na	na	na	na

^a^HER2 IHC: 0–1+ is negative, 2+ is equivocal, 3+ is positive

^b^All patients who received trastuzumab-based treatment received trastuzumab (H) for 1 year unless otherwise specified

^c^Follow up time defined as time in months from date of initial breast biopsy to date of last clinical follow-up

^d^ HER2 ratio 1.0 because HER2 copies and CEP 17 copies both > 20, so HER2 alternative probe ratio performed to confirm HER2 status

*AC* Adriamycin + cyclophosphamide, *AT* adjuvant therapy, *CR* complete response, *ddAC* dose-dense adriamycin + cyclophosphamide, *ER* estrogen receptor, *FISH* fluorescence in-situ hybridization, *H* herceptin (trastuzumab), *HER2* human epidermal growth factor receptor 2, *HER2-E* HER2-enriched, *IDC* invasive ductal cancer, *IHC* immunohistochemistry, *ILC* invasive lobular cancer, *IMPC* invasive micropapillary cancer, *MET* therapy for metastatic disease, *N/A* not applicable, *na* not available, *NAT* neoadjuvant therapy, *NS* not sufficient for testing, *P* pertuzumab, *PR* partial response, *PR* progesterone receptor, *T* weekly paclitaxel ×12, *T-DM1* ado-trastuzumab, *TNM* tumor node metastasis, *Tx* therapy

Among the 31, alternative probe-positive cases, 30 (97%) were ER^+^ and 11 (35%) were high grade. In contrast, among the 19 cases that were HER2^+^ by traditional FISH criteria, nine (47%) were ER^+^ and 14 (74%) were high grade (*p* = 0.0002). Repeat HER2 testing was performed on a second tumor sample in 11 cases and seven of those were negative for HER2 gene amplification by both traditional FISH criteria and alternative probe method. Two cases were confirmed HER2^+^ by conventional FISH and two only by the alternative probe method (Tables 1 and 2).

Adequate tissue for PAM50 intrinsic subtype analysis was available for 20 alternative probe-positive cases and nine cases that were HER2^+^ by traditional FISH criteria. None (0%) of the 20 alternative probe cancers had HER2-enriched intrinsic subtype, while eight (89%) of the nine HER2^+^ traditional controls were of the HER2-enriched molecular subtype (*p* = 0.0001).

Clinical and pathologic data for patients in the alternative probe-positive cohort is provided in Table 1. Staging information was available for 24 patients. Twenty-one had early-stage disease (12 = node negative, 9 = node positive), one patient had a postmastectomy recurrence, and two patients had metastatic disease. Treatment information was available for 22 patients. Only four patients did not receive HER2-targeted therapy, one with metastatic disease and three with node-negative early-stage disease. Of the early stage patients who did receive HER2-targeted therapy, only two received it in the neoadjuvant setting, along with chemotherapy; one had a pathologic complete response (pCR) and one had partial responses (PR). Similarly, only four patients in the traditional HER2^+^ cohort received neoadjuvant HER2-targeted therapy with chemotherapy; two had a pathologic PR and two (both patients with HER2-enriched subtype) achieved pCR (Table 2). Given the small number of patients treated in the neoadjuvant setting, we cannot draw meaningful conclusions regarding response to HER2-targeted therapy among the alternative probe cases.

In the alternative probe-positive cohort, when considering the early-stage patients who received concurrent chemotherapy and HER2-targeted therapy, all except one had ER^+^ tumors, eight had no nodal involvement, and five had only N1a or N1mic disease. Had these tumors been considered HER2-negative, all might have been candidates for genomic expression assays to determine the need for chemotherapy, yet only three were evaluated for such (Table 1). Oncotype Dx was performed on tumors from three patients, with recurrence scores of 29, 26, and 12. The patient with the recurrence score of 12 had T1cN0 ER^+^ disease, was treated with endocrine therapy only without any chemotherapy or HER2-targeted therapy, and remains in remission at the last follow-up.

In conclusion, these findings highlight the unique pathologic and molecular characteristics of breast cancers classified as HER2^+^ only by an alternative probe method, and raise questions regarding the appropriate management of these cancers. More data regarding response of these breast cancers to HER2-targeted therapies is needed.
